# Editorial: Diet, food allergy, and gut immunity

**DOI:** 10.3389/fnut.2023.1276929

**Published:** 2023-08-23

**Authors:** Qiaozhi Zhang, Yinghua Liu, Guangming Liu, Linglin Fu

**Affiliations:** ^1^Food Safety Key Laboratory of Zhejiang Province, School of Food Science and Biotechnology, Zhejiang Gongshang University, Hangzhou, China; ^2^Nutrition Department of the First Medical Centre of PLA General Hospital, Beijing, China; ^3^Fujian Collaborative Innovation Center for Exploitation and Utilization of Marine Biological Resources, College of Food and Biological Engineering, Jimei University, Xiamen, China

**Keywords:** food allergy, food allergen, host immunity, immunomodulatory compound, immunotherapy

Food allergy is a public health issue that receives increasing attention worldwide (1). Defined as a group of IgE-mediated and non-IgE-mediated “adverse immune reactions” that occurs reproducibly upon exposure to a given food, the multifactorial nature of food allergy calls for a deeper understanding of the cross-talk between diet, gut immunity, and the etiology of food allergy that would help to inform the development of effective strategies to reduce the disease burden.

This Research Topic aims to gain new insights into the role of dietary factors and gut homeostasis in maintaining oral tolerance and to provide relevant targets for designing hypoallergic systems for food allergy. Understanding the links between diet, intestinal immunity, and food allergic responses would formulate efficacious prevention and treatment options for food allergy as well as benefiting other atopic disorders.

In this special Research Topic, there are five papers discussing the above-mentioned aspects.

In addition to genetic predisposition, external exposures such as dietary factors are suggested to be the predominant forces driving the increased prevalence of food allergy (2). Ma et al. reported an interesting study by analyzing changes in early-life risk factors for childhood food sensitization during 2009–2019 in Chongqing, China. In addition to family history of atopic diseases, common lifestyle factors in particular feeding patterns are closely associated with the incidence of food sensitization, despite not significantly modified over a period of 10 years. Interestingly, the influence of demographic features, including gender, age and the presence of siblings changed in the past decade and should be considered as modifiable factors in future epidemiological studies.

Considering cow's milk allergy (CMA) as the most prevalent cause of food allergy in infants, Wilsey et al. reported an extensive prospective study examining the short-term symptomatic improvement of suspected CMA by using amino acid formulas (AAF). After 3–6 weeks of AAF initiation, a diverse array of CMA symptoms including gastrointestinal, skin, respiratory as well as uncategorized symptoms were improved to various extents, confirming that AAF can be an effective option for the management of CMA in infancy.

Compared to CMA that majorly affects infants younger than 1 year old and outgrown by the age of three to five, some other types of food allergy such as peanut, seafood and fruit allergy may persist life-long (3). Allergen-specific immunotherapy (AIT) complies an efficacious and safe strategy for mitigating food-allergic symptoms, while the underlying immunogenic events related to tolerant induction need to be fully understood. Núñez et al. conducted an in-depth study on the epigenetic changes of dendric cells (DCs) in a Pru p 3 anaphylactic mouse model in the context of sublingual AIT using an adjuvant mannose dendron-conjugated allergen. Different doses of AIT resulted in different immune responses in the model, as reflected in the induction of various DNA methylation changes in lymph nodes-DCs, mostly those linked to immune and tolerance responses such as T cell and B cell immunity, and effector cytokines. Use of these biomarkers may be of interest for further prediction of the long-lasting consequences of AIT treatments.

Besides AIT, allergen-non-specific compounds with immunomodulatory activities can be also potent to induce oral tolerance and prevent allergy. Two reviews focused on this point and respectively discussed the potential of breast milk microRNAs (miRNAs) and plant polyphenols as anti-allergic components to manage food allergy. Ahlberg et al. summarized the most abundant miRNAs in human milk and predicted their target genes and pathways with bioinformatic tools. The enriched pathways were found to be connected to several key signaling pathways involved in the immune system, which included the TGF-βsignaling, T cell receptor signaling, Jak-STAT signaling, and Th1 and Th2 cell differentiation. Despite functional experiments needed, the predicted implication of breast milk miRNAs highlighted their role in the regulation of tolerogenic responses that confer infants protections against immune diseases including food allergy. On the other hand, polyphenols are a group of secondary metabolites from plants that possess a wide range of biological activities including immunomodulatory and anti-allergic activities (4). Wu et al. reviewed the updated advances in the allergy-preventing effects of plant polyphenols by summarizing relevant researches conducted in cells and animal models. Further application of plant-derived polyphenols as anti-allergic drugs require precise understanding of their molecular mechanisms of action, structure-activity relationship along with their optimum pharmaceutical formulations to avoid any immunotoxic or cross-reactive effects.

In summary, the above-mentioned studies and reviews provided an extended knowledge of the above topics in food allergy. Reading this Research Topic would inform new understanding of the etiology of food allergy in relevance to the connections between dietary factors and immune regulations, reinforcing the belief that the path ahead toward resolving food allergy remains encouraging.

## Author contributions

QZ: Writing—original draft. YL: Writing—review and editing. GL: Writing—review and editing. LF: Writing—review and editing.
